# Retraction: Xanthogranulomatous Cholecystitis: A Rare Variant of Chronic Cholecystitis

**DOI:** 10.7759/cureus.r134

**Published:** 2024-01-25

**Authors:** Raghad K Alammari, Alanoud A Alhessan, Abdulaziz A Alturki, Safa A Aburowais, Mansour H Alsharif, Feras H Alshehri, Feddah M Hakami, Thawab M Alsubaie, Sukaina A Alhamed, Mosab A Alsobhi, Hussain A Alshaqaqiq, Ali I Alshaqaqiq, Abdullah H Alkharraz, Ali M Alhudhayf, Faisal Al-Hawaj

**Affiliations:** 1 Medicine, Almaarefa University, Riyadh, SAU; 2 Medicine, Arabian Gulf University, Manama, BHR; 3 Medicine, Batterjee Medical College, Jeddah, SAU; 4 Medicine, Imam Mohammad Ibn Saud Islamic University, Riyadh, SAU; 5 Medicine, Jazan University, Jazan, SAU; 6 Medicine, Jordan University of Science And Technology, Irbid, JOR; 7 Medicine, King Abdulaziz University, Jeddah, SAU; 8 Medicine, King Faisal University, Hofuf, SAU; 9 Medicine, Qassim University, Buraydah, SAU; 10 College of Medicine, Imam Abdulrahman Bin Faisal University, Dammam, SAU

The Editors-in-Chief have retracted this article. Concerns were raised regarding the identity of the authors on this article. Specifically, Faisal Alhaway and Malak Shammari have stated that they were added to this article without their knowledge or approval. The identity of the other authors could also not be verified. As the appropriate authorship of this work cannot be established, the Editors-in-Chief no longer have confidence in the results and conclusions of this article.

